# Supplementary material for: Exploring Health Behaviours, Attitudes and Beliefs of Women and Men during the Preconception and Interconception Periods: A Cross-Sectional Study of Adults on the Island of Ireland
Source: Nutrients. 2023 Sep 1;15(17):3832. doi: 10.3390/nu15173832 (PMC10490475; doi:10.3390/nu15173832)
Supplement: Supplementary file 1 [file nutrients-15-03832-s001.zip › nutrients-2581186-supplementary.pdf]

Supplementary Material S1.

Questionnaire.

Planning for a Healthy Pregnancy Survey

Start of Block: Consent

Q1 What do men and women aged 18-60 years think about health practices before trying for a baby? We'd like to invite you to take part in our questionnaire-based research study. This works best on Google Chrome, Safari or Firefox. **Participation is entirely voluntary.** Before you decide, we would like you to understand why the research is being carried out and what it would involve for you. Take time to read the information below and please feel free to talk to others about the study if you wish.

Why is the research being carried out? The research is designed to better understand men and women's attitudes towards preconception (or pre-pregnancy) health and lifestyle behaviours (including diet, weight, physical activity, taking vitamin supplements, alcohol, stress, sleep and smoking etc.). **By preconception we mean at least 3 months before trying to become pregnant (not when actively trying for a baby, or when already pregnant).** We are also interested in your current health and lifestyle behaviours. We are looking for both men and women to take part in the survey and you don't have to have already had a pregnancy (planned or unplanned), or have children to take part - we are interested in everyone's views.

Why have I been asked to take part and am I eligible? We are seeking men and women of child-bearing age across the UK to participate, who are between the ages of 18-60 years (with or without children).

Who can take part? Anyone (male or female) who is aged 18-60 years, with or without children or currently pregnant.

Purpose of the research: The purpose of this study is to seek the views of both men and women in relation to preconception (or pre-pregnancy) lifestyle behaviours, and current health and lifestyle behaviours. In other words, we want to understand more about peoples' views on diet, weight, physical activity, taking vitamin supplements, smoking, alcohol, stress and sleep before trying for a baby or becoming pregnant, and also how they currently behave in relation to these areas.

What does it involve? 1. You will be asked to complete a consent statement after reading this information. 2. You will take part in a one-off questionnaire which includes questions on your personal background (age, ethnicity, household, education, marital status, medical conditions etc.) alongside your attitudes towards preconception (or pre-pregnancy) health and lifestyle behaviours including diet, activity, smoking, alcohol, stress etc., as well as some questions on your current lifestyle behaviours. 3. Please feel free to leave blank any questions you do not feel comfortable answering by skipping to the next question. 4. All data will be anonymous, we will not ask for your name or contact details and all information will be held in accordance with the data protection act. 5. We believe it will take about 15 minutes of your time.

Who is organising and funding the research? This study is part of a 3rd year student project being conducted at the Centre for Public Health, Queen's University Belfast.

Who has reviewed this study? The study has been reviewed and approved by Queen's University Belfast School of Medicine, Dentistry and Biomedical Sciences Research Ethics Committee.

Who do I contact if I have any questions? Please contact the research team by e-mailing Dr Laura McGowan at: laura.mcgowan@qub.ac.uk

After reading the participant information sheet (above), you must complete the consent process before taking part. if you want to continue with the questionnaire, <u>tick 'Yes' to all</u> the following statements:

	Yes (1)	No (2)
I CONFIRM THAT I HAVE READ AND UNDERSTAND THE INFORMATION SHEET RELATED TO THIS STUDY. I HAVE HAD THE OPPORTUNITY TO ASK QUESTIONS (VIA EMAILING THE RESEARCH TEAM) AND THESE HAVE BEEN ANSWERED FULLY. (1)	○	○
I UNDERSTAND THAT MY PARTICIPATION IS VOLUNTARY AND I AM FREE TO WITHDRAW/EXIT THE SURVEY AT ANY TIME, WITHOUT GIVING ANY REASON AND WITHOUT MY LEGAL RIGHTS OR MEDICAL CARE BEING AFFECTED. I CAN ALSO OMIT ANY QUESTONS I WISH. (2)	○	○
I UNDERSTAND ALL DATA WILL BE TREATED SECURELY AS DESCRIBED BY THE DATA PROTECTION ACT (1998) AND STORED APPROPRIATELY AS REQUIRED BY THE UNIVERSITY. (3)	○	○
I AM AWARE OF THAT ALL OF MY INFORMATION WILL BE TREATED AS ANONYMOUS AND CONFIDENTIAL, AND I WOULD NOT BE PERSONALLY IDENTIFIABLE FROM ANY OF THE RESEARCH. (4)	○	○
I HAVE BEEN GIVEN CONTACT INFORMATION FOR THE RESEARCHERS. (5)	○	○
I AGREE TO TAKE PART IN THIS SURVEY RESEARCH STUDY. (6)	○	○

Q2 **Thank you for taking the time to participate in this questionnaire. Please do the best you can to answer all the questions, however, if there are any questions you do not wish to answer feel free to leave them out without giving us a reason.**

End of Block: Consent

Start of Block: Demographics

Q3 How old are you?

Age (1)

▼ 18 (0) ... 60 (42)

Q4 What is your ethnicity?

- ○ White (1)
- ○ Irish Traveller (2)
- ○ White and Black Caribbean (3)
- ○ White and Black African (4)
- ○ White and Asian (5)
- ○ Other Mixed Multiple ethnic background, please specify: (6) ____
- ○ Asian British (7)
- ○ Indian (8)
- ○ Pakistani (9)
- ○ Bangladeshi (10)
- ○ Chinese (11)
- ○ Other Asian background, please specify: (12) ____
- ○ African (13)
- ○ Caribbean (14)
- ○ Black British (15)
- ○ Other Black / African / Caribbean background, please specify: (16) ____

- Arab (17)
- Other ethnic group Arab, please specify: (18) ________________________________

Q5 What is your country of residence? (Mark one only)

- Northern Ireland (1)
- Republic of Ireland (2)
- Scotland (3)
- Wales (4)
- England (5)
- Other – please state: (6) ________________________________

Q6 What sex are you?

- Male (1)
- Female (2)
- Other (3)
- Prefer not to say (4)

Q7 Are you currently in a relationship?

- Yes (1)
- No (2)

Q8 Do you currently have any children (biological or otherwise)?

- Yes (1)
- No (2)

Q9 Are you or your partner currently pregnant?

- Yes (1)
- No (2)
- Not Applicable (3)

Q10 Have you or a partner (current or past) been pregnant before (regardless of pregnancy outcome)?

- Yes (1)
- No (2)
- Not Applicable (3)

Skip To: Q14 If Have you or a partner (current or past) been pregnant before (regardless of pregnancy outcome)? = No

Skip To: Q11 If Have you or a partner (current or past) been pregnant before (regardless of pregnancy outcome)? = Yes

Skip To: Q14 If Have you or a partner (current or past) been pregnant before (regardless of pregnancy outcome)? = Not Applicable

---

Display This Question:

If Have you or a partner (current or past) been pregnant before (regardless of pregnancy outcome)? = Yes

Q11 How many times have you/your partner been previously pregnant?

(1)

▼ 1 (0) ... 50 (49)

Display This Question:

If Have you or a partner (current or past) been pregnant before (regardless of pregnancy outcome)? = Yes

Or Are you or your partner currently pregnant? = Yes

Q12 Did you or that partner plan to become pregnant?

- ◯ On all occasions (1)
- ◯ On most occasions (2)
- ◯ Did not plan to get pregnant (3)

Q13 Do you intend to try for a baby (first baby, or additional children)? Please choose one:

- ◯ No (1)
- ◯ Not sure (2)
- ◯ Yes - in the next year (3)
- ◯ Yes - in the next 1 - 5 years (4)
- ◯ Yes - in the next 6 - 10 years (5)
- ◯ Yes - in the next 10+ years (6)

Display This Question:

If Are you or your partner currently pregnant? != Yes

And Have you or a partner (current or past) been pregnant before (regardless of pregnancy outcome)? != Yes

Q14 If you have <u>never</u> experienced a pregnancy (personally or via a partner), do you think you would plan for a pregnancy by changing your diet, physical activity, smoking or alcohol etc.?

- ◯ Yes (1)
- ◯ No (2)
- ◯ Not sure (3)

End of Block: Demographics

Start of Block: Medical

Q15 Do you suffer from any medical conditions? Tick all apply

- ▢ **No- I do not suffer from any medical conditions** (436)
- ▢ Diabetes T1 (437)
- ▢ Diabetes T2 (438)
- ▢ Hypertensive disorders (blood pressure) (439)
- ▢ Congenital or acquired cardiac disease (440)
- ▢ Autoimmune disorders (441)
- ▢ Renal or liver disease (442)
- ▢ Reflux disease (GERD) (443)
- ▢ Irritable bowel syndrome (444)
- ▢ Inflammatory bowel disease (445)
- ▢ HIV infection (446)
- ▢ Obesity (447)
- ▢ Epilepsy (448)
- ▢ Any mental health problem (449)
- ▢ Stress (450)
- ▢ Anxiety (451)
- ▢ Depression (452)
- ▢ Asthma (453)

▢ Other - please specify: (454) ____

Display This Question:

If Are you or your partner currently pregnant? = Yes

And Do you suffer from any medical conditions? Tick all apply != No- I do not suffer from any medical conditions

Or Have you or a partner (current or past) been pregnant before (regardless of pregnancy outcome)? = Yes

And Do you suffer from any medical conditions? Tick all apply != No- I do not suffer from any medical conditions

Q16 For your or your partner's current or past pregnancy pregnancy, did you seek or receive any specific medical advice about your medical condition regarding pregnancy?

○ Yes (1)

○ No (2)

End of Block: Medical

Start of Block: SECTION 2: ATTITUDES TOWARDS HEALTH/LIFESTYLE BEHAVIOURS PRE-PRECONCEPTION

Q17 The following questions ask about a range of lifestyle behaviours relating to both men and women in the preconception period i.e. before trying for a baby.
By preconception we mean at least 3 months before trying to become pregnant (not when actively trying for a baby, or when already pregnant).
Please indicate how much you agree or disagree with the following statements. Choose one for each.

Q18 It is important for **women** to consume a healthy diet **preconception** (i.e. one that is high in fruit and vegetables and wholegrains, and low in fat, sugar and salt).

- ◯ Strongly disagree (1)
- ◯ Somewhat disagree (2)
- ◯ Neither agree nor disagree (3)
- ◯ Somewhat agree (4)
- ◯ Strongly agree (5)

Q19 It is important for **men** to consume a healthy diet **preconception** (i.e. one that is high in fruit and vegetables and wholegrains, and low in fat, sugar and salt).

- ◯ Strongly disagree (1)
- ◯ Somewhat disagree (2)
- ◯ Neither agree nor disagree (3)
- ◯ Somewhat agree (4)
- ◯ Strongly agree (5)

Q20 It is important for **women** to take folic acid **preconception**.

- ◯ Strongly disagree (1)
- ◯ Somewhat disagree (2)
- ◯ Neither agree nor disagree (3)
- ◯ Somewhat agree (4)
- ◯ Strongly agree (5)

Q21 It is important for **women** to take vitamin D **preconception.**

- Strongly disagree (1)
- Somewhat disagree (2)
- Neither agree nor disagree (3)
- Somewhat agree (4)
- Strongly agree (5)

Q22 It is important for **men** to take vitamins **preconception**, such as vitamin D.

- Strongly disagree (1)
- Somewhat disagree (2)
- Neither agree nor disagree (3)
- Somewhat agree (4)
- Strongly agree (5)

Q23 Remember: By preconception (pre-pregnancy) we mean at least 3 months before trying to become pregnant (not when actively trying for a baby, or when already pregnant).

Q24 It is important for **women** to achieve a healthy weight **preconception.**

- Strongly disagree (1)
- Somewhat disagree (2)
- Neither agree nor disagree (3)
- Somewhat agree (4)
- Strongly agree (5)

Q25 It is important for **men** to achieve a healthy weight **preconception**.

- Strongly disagree (1)
- Somewhat disagree (2)
- Neither agree nor disagree (3)
- Somewhat agree (4)
- Strongly agree (5)

Q26 It is important for **women** to be physically active **preconception** (i.e. at least 2.5 hours/150 minutes of moderate aerobic activity e.g. cycling/brisk walking weekly, plus resistance exercises at least twice per week).

- Strongly disagree (1)
- Somewhat disagree (2)
- Neither agree nor disagree (3)
- Somewhat agree (4)
- Strongly agree (5)

Q27 It is important for **men** to be physically active **preconception** (i.e. at least 2.5 hours/150 minutes of moderate aerobic activity e.g. cycling/brisk walking weekly, plus resistance exercises at least twice per week).

- Strongly disagree (1)
- Somewhat disagree (2)
- Neither agree nor disagree (3)
- Somewhat agree (4)
- Strongly agree (5)

Q28 Remember: By <u>preconception</u> (pre-pregnancy) we mean at least 3 months <u>before trying</u> to become pregnant (not when actively trying for a baby, or when already pregnant).

Q29 It is important for **women** to stop smoking cigarettes **<u>preconception</u>**.

- ○ Strongly disagree (1)
- ○ Somewhat disagree (2)
- ○ Neither agree nor disagree (3)
- ○ Somewhat agree (4)
- ○ Strongly agree (5)

Q30 It is important for **men** to stop smoking cigarettes **<u>preconception</u>**.

- ○ Strongly disagree (1)
- ○ Somewhat disagree (2)
- ○ Neither agree nor disagree (3)
- ○ Somewhat agree (4)
- ○ Strongly agree (5)

Q31 It is important for **women** to stop using e-cigarettes **<u>preconception</u>**.

- ○ Strongly disagree (1)
- ○ Somewhat disagree (2)
- ○ Neither agree nor disagree (3)
- ○ Somewhat agree (4)
- ○ Strongly agree (5)

Q32 It is important for **men** to stop using e-cigarettes **preconception**.

- Strongly disagree (1)
- Somewhat disagree (2)
- Neither agree nor disagree (3)
- Somewhat agree (4)
- Strongly agree (5)

Q33 Remember: By preconception (pre-pregnancy) we mean at least 3 months before trying to become pregnant (not when actively trying for a baby, or when already pregnant).

Q34 It is important for **women** to stop drinking alcohol **preconception**.

- Strongly disagree (1)
- Somewhat disagree (2)
- Neither agree nor disagree (3)
- Somewhat agree (4)
- Strongly agree (5)

Q35 It is important for **men** to stop drinking alcohol **preconception**.

- Strongly disagree (1)
- Somewhat disagree (2)
- Neither agree nor disagree (3)
- Somewhat agree (4)
- Strongly agree (5)

Q36 Remember: By preconception (pre-pregnancy) we mean at least 3 months before trying to become pregnant (not when actively trying for a baby, or when already pregnant).

Q37 It is important for **women** to get at least 7-8 hours of sleep per night **preconception**.

- Strongly disagree (1)
- Somewhat disagree (2)
- Neither agree nor disagree (3)
- Somewhat agree (4)
- Strongly agree (5)

Q38 It is important for **men** to get at least 7-8 hours of sleep per night **preconception**.

- Strongly disagree (1)
- Somewhat disagree (2)
- Neither agree nor disagree (3)
- Somewhat agree (4)
- Strongly agree (5)

Q39 It is important for **women** to take steps to reduce stress in their daily lives **preconception**.

- Strongly disagree (1)
- Somewhat disagree (2)
- Neither agree nor disagree (3)
- Somewhat agree (4)
- Strongly agree (5)

Q40 It is important for **men** to take steps to reduce stress in their daily lives **preconception**.

- Strongly disagree (1)
- Somewhat disagree (2)
- Neither agree nor disagree (3)
- Somewhat agree (4)
- Strongly agree (5)

Q41 Remember: By preconception (pre-pregnancy) we mean at least 3 months before trying to become pregnant (not when actively trying for a baby, or when already pregnant).

Q42 It is important for **women** to visit a healthcare professional **preconception to discuss health practices**.

- Strongly disagree (1)
- Somewhat disagree (2)
- Neither agree nor disagree (3)
- Somewhat agree (4)
- Strongly agree (5)

Q43 It is important for **men** to visit a healthcare professional **preconception to discuss health practices.**

- Strongly disagree (1)
- Somewhat disagree (2)
- Neither agree nor disagree (3)
- Somewhat agree (4)
- Strongly agree (5)

Display This Question:

If What sex are you? = Female

Q44 **In your opinion,** please rank the importance of the following lifestyle changes **for women preconception, i.e. before trying for a baby** – with 1 being most important to 12 being least important (drag the options into order and, if on mobile, the **far right edge of the screen can be used to scroll**):

______ Consume a healthy diet (1)
______ Achieve a healthy weight (2)
______ Take folic acid (3)
______ Take vitamin D (4)
______ Be physically active in line with national recommendations (5)
______ Stop smoking cigarettes (6)
______ Stop using e-cigarettes (7)
______ Cut out alcohol (8)
______ Actively reduce/manage stress (9)
______ Address mental health issues (10)
______ Get adequate amounts of sleep (11)
______ Visit a healthcare professional (including medication/drugs review, STI screening, immunisations etc.) (12)

Display This Question:

If What sex are you? = Male

Q45 In your opinion, please rank the importance of the following lifestyle changes for men preconception, i.e. before trying for a baby – with 1 being most important to 11 being least important (drag the options into order and, if on mobile, the **right edge of the screen can be used to scroll**):

______ Consume a healthy diet (1)
______ Achieve a healthy weight (2)
______ Take vitamin D (3)
______ Be physically active in line with national recommendations (4)
______ Stop smoking cigarettes (5)
______ Stop using e-cigarettes (6)
______ Cut out alcohol (7)
______ Actively reduce/manage stress (8)
______ Address mental health issues (9)
______ Get adequate amounts of sleep (10)
______ Visit a healthcare professional (including medication/drugs review, STI screening, immunisations etc.) (11)

Q46 The statements below address your attitudes to preconception health and lifestyle care. **By preconception (pre-pregnancy) we mean at least 3 months before trying to become pregnant (not when actively trying for a baby, or when already pregnant).**

Q47 To what extent do you agree with the following statements?

	Strongly Disagree (1)	Disagree (2)	Neither Agree or Disagree (3)	Agree (4)	Strongly Agree (5)
Preconception care does not have any effect on pregnancy outcome (1)	○	○	○	○	○
Preconception care is an important health issue for women of child bearing age (2)	○	○	○	○	○
Preconception care is an important health issue for men of child bearing age (3)	○	○	○	○	○
A dedicated clinic for preconception care is something I would make use of, if available (4)	○	○	○	○	○
My friends/family do not plan for pregnancy (5)	○	○	○	○	○
Preconception care is a high priority for me (6)	○	○	○	○	○
There is little evidence base for preconception care (7)	○	○	○	○	○
My GP is an appropriate person to offer preconception advice (8)	○	○	○	○	○

My practice nurse or community midwife is an appropriate person to offer preconception advice (9)	○	○	○	○	○
Friends and family are the best place to get preconception care information (10)	○	○	○	○	○
The internet is an appropriate place to get preconception advice (11)	○	○	○	○	○

Display This Question:

If What sex are you? = Female

Q48 For the following health practices, please consider the statement:

"Improving my health, preconception, in the area of ...X... is mainly for the benefit of *my personal health* or *my potential baby's health* etc."

	My personal health (1)	My potential baby's health (2)	Both (3)	Neither (4)	Not sure (5)
...Healthy diet (1)	○	○	○	○	○
...Healthy weight (2)	○	○	○	○	○
...Physically active (3)	○	○	○	○	○
...Folic acid (4)	○	○	○	○	○
...Vitamin D (5)	○	○	○	○	○
...Stopping smoking cigarettes (6)	○	○	○	○	○
...Stopping use of e-cigarettes (7)	○	○	○	○	○
...Cutting out alcohol (8)	○	○	○	○	○
...Reducing stress (9)	○	○	○	○	○
...Getting adequate sleep (7-8 hrs) (10)	○	○	○	○	○
...Seeing a healthcare professional (11)	○	○	○	○	○

Display This Question:

If What sex are you? = Male

Q49 For the following health practices, please consider the statement:

"Improving my health, <u>preconception</u>, in the area of ...X... is mainly for the benefit of *my personal health* <u>or</u> *my potential baby's health* etc."

	My personal health (1)	My potential baby's health (2)	Both (3)	Neither (4)	Not sure (5)
...Healthy diet (1)	o	o	o	o	o
...Healthy weight (2)	o	o	o	o	o
...Physically active (3)	o	o	o	o	o
...Vitamin D (4)	o	o	o	o	o
...Stopping smoking cigarettes (5)	o	o	o	o	o
...Stopping use of e-cigarettes (6)	o	o	o	o	o
...Cutting out alcohol (7)	o	o	o	o	o
...Reducing stress (8)	o	o	o	o	o
...Getting adequate sleep (7-8 hrs) (9)	o	o	o	o	o
...Seeing a healthcare professional (10)	o	o	o	o	o

End of Block: SECTION 2: ATTITUDES TOWARDS HEALTH/LIFESTYLE BEHAVIOURS PRE-PRECONCEPTION

Start of Block: Section 3- PERSONALLY HAD A PREVIOUS PREGNANCY

Q50 **Please think of your <u>current or most recent pregnancy</u> (if more than one) when answering the following section.**

Q51 In the month that I became pregnant… (Please tick the statement which <u>most</u> applies to you):

- ◯ I/ we were not using contraception (1)
- ◯ I/ we were using contraception, but not on every occasion (2)
- ◯ I/ we always used contraception, but knew that the method had failed (i.e., broke, moved, came off, came out, not worked etc) at least once (3)
- ◯ I/ we always used contraception (4)

Q52 In terms of becoming a mother *(first time or again)*, I feel that the pregnancy happened at the… (Please tick the statement which <u>most</u> applies to you):

- ◯ Right time (1)
- ◯ Ok, but not quite the right time (2)
- ◯ Wrong time (3)

Q53 Just <u>before</u> I became pregnant… (Please tick the statement which <u>most</u> applies to you):

- ◯ I intended to get pregnant (1)
- ◯ My intentions kept changing (2)
- ◯ I did not intend to get pregnant (3)

Q54 Just <u>before</u> I became pregnant… (Please tick the statement which <u>most</u> applies to you):

- ◯ I wanted to have a baby (1)
- ◯ I had mixed feelings about having a baby (2)
- ◯ I did not want to have a baby (3)

Q55 Before I became pregnant… (Please tick the statement which most applies to you):

- ◯ My partner and I had agreed that we would like me to be pregnant (1)
- ◯ My partner and I had discussed having children together, but hadn't agreed for me to get pregnant (2)
- ◯ We never discussed having children together (3)

Q56 Did you make any changes to your lifestyle in preparation for becoming pregnant? (Please tick all that apply):

- ▢ Took folic acid (1)
- ▢ Took a multivitamin or other vitamin supplement (2)
- ▢ Stopped or cut down smoking (3)
- ▢ Stopped or cut down drinking alcohol (4)
- ▢ Ate more healthily (5)
- ▢ Drank an adequate amount of fluids (e.g. water) (6)
- ▢ Started or maintained being physically active (7)
- ▢ Achieved a healthy weight (8)
- ▢ Sought medical/ health advice relating to conception/pregnancy (9)
- ▢ Took steps to reduce stress levels (10)
- ▢ Got an adequate amount of sleep (7-8 hours per night) (11)
- ▢ Cut down on caffeinated beverages (two or less per day) (12)
- ▢ Took vitamin D (13)
- ▢ Stopped using recreational drugs (14)
- ▢ Took some other action, please describe: (15) ____
- ▢ I did not do any of the above before my pregnancy (16)

Q57 Did you conceive:

- Naturally (1)
- With assisted reproduction (e.g. IVF/ ICSI treatment) (2)

Q58 How long did it take you to conceive?

- 0-6 months (1)
- 6-12 months (2)
- Longer than 12 months (3)
- I did not plan to fall pregnant (4)

End of Block: Section 3- PERSONALLY HAD A PREVIOUS PREGNANCY

Start of Block: section 3-PARTNER (CURRENT OR PAST) HAD A PREVIOUS PREGNANCY

Q59 **Please think of your current or past partner's present or most recent pregnancy (if more than one) when answering the following section.**

Q60 In the month that my partner became pregnant… (Please tick the statement which most applies to you):

- I/ we were not using contraception (1)
- I/ we were using contraception, but not on every occasion (2)
- I/ we always used contraception, but knew that the method had failed (i.e., broke, moved, came off, came out, not worked etc) at least once (3)
- I/ we always used contraception (4)

Q61 In terms of becoming a parent *(first time or again)*, I feel that the pregnancy happened at the… (Please tick the statement which most applies to you):

- ○ Right time (1)
- ○ Ok, but not quite the right time (2)
- ○ Wrong time (3)

Q62 Just before my partner became pregnant… (Please tick the statement which most applies to you):

- ○ I intended for my partner to get pregnant (1)
- ○ My intentions kept changing (2)
- ○ I did not intend for my partner to get pregnant (3)

Q63 Just before my partner became pregnant… (Please tick the statement which most applies to you):

- ○ I wanted to have a baby (1)
- ○ I had mixed feelings about having a baby (2)
- ○ I did not want to have a baby (3)

Q64 Before my partner became pregnant… (Please tick the statement which most applies to you):

- ○ My partner and I had agreed that we would like her to be pregnant (1)
- ○ My partner and I had discussed having children together, but hadn't agreed for her to get pregnant (2)
- ○ We never discussed having children together (3)

Q65 Did you make any changes to your lifestyle in preparation for your partner becoming pregnant? (Please tick all that apply):

- ☐ Took a multivitamin or other vitamin supplement (1)
- ☐ Stopped or cut down smoking (2)
- ☐ Stopped or cut down drinking alcohol (3)
- ☐ Ate more healthily (4)
- ☐ Drank an adequate amount of fluids (e.g. water) (5)
- ☐ Started or maintained being physically active (6)
- ☐ Achieved a healthy weight (7)
- ☐ Sought medical/ health advice relating to conception/pregnancy (8)
- ☐ Took steps to reduce stress levels (9)
- ☐ Got an adequate amount of sleep (7-8 hours per night) (10)
- ☐ Cut down on caffeinated beverages (two or less per day) (11)
- ☐ Took vitamin D (12)
- ☐ Stopped using recreational drugs (13)
- ☐ Took some other action, please describe: (14) ____
- ☐ I did not do any of the above before my partner's pregnancy (15)

Q66 Did you conceive:

- ○ Naturally (1)
- ○ With assisted reproduction (e.g. IVF treatment) (2)

Q67 How long did it take to conceive?

- ○ 0-6 months (1)
- ○ 6-12 months (2)
- ○ Longer than 12 months (3)
- ○ I did not plan to fall pregnant (4)

End of Block: section 3-PARTNER (CURRENT OR PAST) HAD A PREVIOUS PREGNANCY

Start of Block: Section 4-SOURCES OF ADVICE/INFORMATION ON PRECONCEPTION HEALTH

Q68 Have you ever researched information about the following health and lifestyle topics relevant to planning a pregnancy e.g. – (tick all that apply)

- ▢ Healthy diet (1)
- ▢ Healthy weight (2)
- ▢ Safe physical activity (3)
- ▢ Alcohol consumption (4)
- ▢ Smoking (5)
- ▢ Immunisations (6)
- ▢ Recreational drug use (7)
- ▢ STI check (8)
- ▢ Dental health (9)
- ▢ Caffeine consumption (10)
- ▢ Stopping contraception (11)
- ▢ Folic acid and vitamin supplements (12)
- ▢ Stress management (13)
- ▢ Adequate sleep (14)
- ▢ Other – please state: (15) ____
- ▢ I have never looked up any of this information (16)

Display This Question:

If Have you ever researched information about the following health and lifestyle topics relevant to... != I have never looked up any of this information

Q69 Where did you look for this information?

- ☐ Health professionals – e.g. GP, midwife, practice nurse etc. (1)
- ☐ Family or friends (2)
- ☐ Online (3)
- ☐ Library / book / magazine etc. (4)
- ☐ Leaflet e.g. in GP surgery (5)
- ☐ Dedicated preconception clinic (6)
- ☐ Other – please specify: (7) ____

Q70 Have you ever seen information advertised about the following health and lifestyle topics relevant to planning a pregnancy e.g. – (tick all that apply)

- ☐ Healthy diet (1)
- ☐ Healthy weight (2)
- ☐ Safe physical activity (3)
- ☐ Alcohol consumption (4)
- ☐ Smoking (5)
- ☐ Immunisations (6)
- ☐ Recreational drug use (7)
- ☐ STI check (8)
- ☐ Dental health (9)
- ☐ Caffeine consumption (10)
- ☐ Stopping contraception (11)
- ☐ Folic acid and vitamin supplements (12)
- ☐ Stress management (13)
- ☐ Adequate sleep (14)
- ☐ Other – please state: (15) ____
- ☐ I have never seen any of this information advertised (16)

Display This Question:

If Have you ever seen information advertised about the following health and lifestyle topics relevan... != I have never seen any of this information advertised

Q71 Where did you see this information advertised?

- ▢ Health professionals – e.g. GP, midwife, practice nurse etc. (1)
- ▢ Family or friends (2)
- ▢ Online (3)
- ▢ Library/book/magazine etc. (4)
- ▢ Leaflet e.g. in GP surgery (5)
- ▢ Dedicated preconception clinic (6)
- ▢ Other – please specify: (7) ____

Display This Question:

If Have you ever researched information about the following health and lifestyle topics relevant to... = I have never looked up any of this information

Q72 Would you consider looking for preconception healthy lifestyle advice in the future?

- ○ Yes (1)
- ○ No (2)
- ○ Not sure (3)
- ○ Don't think it is important for me (4)

Display This Question:

If Would you consider looking for preconception healthy lifestyle advice in the future? = Yes

Q73 Where would you consider looking?

- ☐ Health professional – e.g. GP, midwife, practice nurse etc. (1)
- ☐ Family or friends (2)
- ☐ Online (3)
- ☐ Library book/magazine etc. (4)
- ☐ Leaflet e.g. in GP surgery (5)
- ☐ Dedicated preconception clinic (6)
- ☐ Other – please specify: (7) ____

End of Block: Section 4-SOURCES OF ADVICE/INFORMATION ON PRECONCEPTION HEALTH

Start of Block: SECTION 5: CURRENT LIFESTYLE BEHAVIOURS

Q74 For the following questions please describe your **current** health and lifestyle practices, even if they are altered because you or your partner is pregnant.

Q75 How often do you consume each of the following? Choose one for each

	Never (1)	Some days but not all days (2)	Everyday (3)
...breakfast (over a week)? (1)	○	○	○
...two portions of fruit per day? (2)	○	○	○
...three portions of vegetables per day? (3)	○	○	○

Q76 What type of bread do you **normally** consume? Choose one only

○ White bread only (1)

○ Sometimes white bread, sometimes brown or granary or wholemeal bread (including 50:50 bread) (2)

○ Only brown/granary bread (including 50:50 bread) (3)

○ Sometimes brown/granary bread (including 50:50 bread), sometimes wholemeal bread (4)

○ Only wholemeal bread (5)

○ I never consume bread (6)

Q77 What type of milk do you **normally** consume? Choose one only

- ○ Whole milk only (1)
- ○ Sometimes whole milk, sometimes semi-skimmed or skimmed milk (2)
- ○ Semi-skimmed milk only (3)
- ○ Sometimes semi-skimmed milk, sometimes skimmed milk (4)
- ○ Skimmed milk only (5)
- ○ Only 1% milk (6)
- ○ Non-dairy alternatives (e.g. soya, almond, rice, etc.) (7)
- ○ I never consume milk (8)

Q78 Do you follow a special diet?

- ○ No (1)
- ○ Yes - self determined e.g. for intolerances (2)
- ○ Yes - prescribed by a medical doctor/dietitian (3)
- ○ Yes - a slimming/weight-loss diet (4)
- ○ Yes - a vegetarian diet (5)
- ○ Yes - a vegan diet (6)

Skip To: Q79 If Do you follow a special diet? = No

Q79 Do you regularly take any vitamins, minerals or other food supplements?

- ○ Yes (1)
- ○ No (2)

Skip To: Q81 If Do you regularly take any vitamins, minerals or other food supplements? = No

Q80 Please specify which vitamins, minerals or other food supplements you take:

____

Q81 **In the past week,** on how many days have you done a total of 30 minutes or more of physical activity, which was enough to raise your breathing rate through sport, exercise etc., but not including housework or physical activity that may be part of your job. Please choose one:

- ◯ 0 (1)
- ◯ 1 (2)
- ◯ 2 (3)
- ◯ 3 (4)
- ◯ 4 (5)
- ◯ 5 (6)
- ◯ 6 (7)
- ◯ 7 (8)

Q82 What is your height? (answer one only)

- ◯ CM (1) ____
- ◯ FT & INCHES (in the form 5,7 etc) (2) ____

Q83 What is your weight? (answer one only)

- ◯ Kg (1) ____
- ◯ ST & LBS (in the form 10,2 etc) (2) ____

Q84 How would you describe your weight? Please mark one only

- ◯ Very underweight (1)
- ◯ Slightly underweight (2)
- ◯ About the right weight (3)
- ◯ Slightly overweight (4)
- ◯ Very overweight (5)

Q85 How many hours per night, on average, are you currently sleeping?

(1)

▼ 1 (0) ... 15 (28)

Q86 Do you consider your current sleep…

- ◯ Poor (1)
- ◯ Average (2)
- ◯ Good (3)
- ◯ Very good (4)

Q87 Do you consider yourself to be under continual stress?

- ◯ Yes (1)
- ◯ No (2)

Q88 Which statement best reflects your smoking status?

- ○ Current smoker (cigarettes) (1)
- ○ Current smoker (e- cigarettes) (2)
- ○ Current smoker (cigarettes AND e-cigarettes) (3)
- ○ Social smoker e.g. on nights out (4)
- ○ Former smoker/quit smoking (5)
- ○ Never smoked (6)

Skip To: Q92 If Which statement best reflects your smoking status? = Never smoked

Display This Question:

If Which statement best reflects your smoking status? = Current smoker (cigarettes)

Or Which statement best reflects your smoking status? = Current smoker (e- cigarettes)

Or Which statement best reflects your smoking status? = Current smoker (cigarettes AND e-cigarettes)

Or Which statement best reflects your smoking status? = Former smoker/quit smoking

Or Which statement best reflects your smoking status? = Social smoker e.g. on nights out

Q89 At what age did you start smoking?

____

Display This Question:

If Which statement best reflects your smoking status? = Current smoker (cigarettes)

Or Which statement best reflects your smoking status? = Current smoker (cigarettes AND e-cigarettes)

Or Which statement best reflects your smoking status? = Former smoker/quit smoking

Q90 How many cigarettes do/did you smoke in an average day?

____

Display This Question:

If Which statement best reflects your smoking status? = Former smoker/quit smoking

Q91 At what age did you stop smoking?

____

Q92 How often do you normally drink alcohol?

- ○ Never (1)
- ○ Occasionally (2)
- ○ Once or twice a week (3)
- ○ Three to five times a week (4)
- ○ Six or seven times a week (5)

Skip To: End of Block If How often do you normally drink alcohol? = Never

Q93 How many **alcoholic drinks**, on average, would you consume **each week**?

(One drink = one regular glass of wine, one measure of spirits, or one half pint of ordinary strength beer)

- ○ None (1)
- ○ 1-10 (2)
- ○ 10-20 (3)
- ○ 20-30 (4)
- ○ More than 30 (5)

End of Block: SECTION 5: CURRENT LIFESTYLE BEHAVIOURS

Start of Block: SECTION 6: EDUCATION, EMPLOYMENT

Q94 What is the highest level of education (full-time or part-time) which you have completed to date?

- No formal education (1)
- Primary education (2)
- Lower secondary: Junior/Intermediate/Group certificate, 'O' Levels/GCSEs, NCVA Foundation Certificate (3)
- Upper secondary: Leaving Certificate (including Applied and Vocational Programmes), 'A' Levels, NCVA (4)
- Technical or Vocational qualification: Completed Apprenticeship, NCVA Level 2/3 Certificate, (5)
- Third Level: Non Degree: National Certificate, Diploma NCEA/Institute of Technology or equivalent, (6)
- Primary Degree (Third Level Bachelor's Degree) (7)
- Professional qualification (of Degree status at least) (8)
- Professional qualification (vocationally trained nurses, police service etc) (9)
- Both a Degree and a Professional Qualification, Postgraduate Certificate or Diploma (10)
- Post-graduate degree (Masters) (11)
- Doctorate (Ph.D.) (12)
- I am still in full-time/ part-time education (13)

Q95 Is your current accommodation:

- ◯ Rented furnished or part-furnished via private rental (1)
- ◯ Rented unfurnished other than from a Local Authority or Voluntary Body (2)
- ◯ Owner occupied - where loan or mortgage repayments are being made (3)
- ◯ Owner occupied - in full with no repayments being made (4)
- ◯ Rented from a Local Authority (welfare provided) (5)
- ◯ Being purchased from a Local Authority under a Tenant Purchase Scheme (6)
- ◯ Rented from a Voluntary Body (7)
- ◯ Occupied free of rent (caretaker, company official, etc.) (8)

Q96 Currently, are you:

- ◯ Full-time employed (1)
- ◯ Part-time employed (2)
- ◯ Self-employed (3)
- ◯ Home-maker (4)
- ◯ Student (5)
- ◯ Unemployed (6)

Display This Question:

If Currently, are you: = Full-time employed

Or Currently, are you: = Part-time employed

Or Currently, are you: = Self-employed

Q97 What is your job title?

____

____

____

____

____

Q98 What is the occupation of the highest income earner in your household?

____

____

____

____

____

End of Block: SECTION 6: EDUCATION, EMPLOYMENT

Supplementary Material S2.

Table S1. Results by age for questions on attitudes towards preconception health and care.

		Agree	Neither Agree or Disagree	Disagree	Statistical test
**Preconception care does not have any effect on pregnancy outcome:**	Age 18-27:	9.00%	12.60%	78.40%	0.491
	Age 28-60:	8.40%	9.00%	82.50%	
**Preconception care is an important health issue for women of childbearing age:**	Age 18-27:	77.60%	17.60%	4.80%	0.040
	Age 28-60:	87.80%	8.50%	3.70%	
**Preconception care is an important health issue for men of childbearing age:**	Age 18-27:	56.00%	30.10%	13.90%	0.167
	Age 28-60:	65.10%	22.90%	12.00%	
**A dedicated clinic for preconception care is something I would make use of, if available:**	Age 18-27:	52.40%	25.90%	21.70%	0.281
	Age 28-60:	46.40%	28.30%	25.30%	
**My friends/family do not plan for pregnancy:**	Age 18-27:	22.90%	37.30%	39.80%	<0.001
	Age 28-60:	11.50%	30.90%	57.60%	
**Preconception care is a high priority for me:**	Age 18-27:	41.00%	26.50%	32.50%	0.009
	Age 28-60:	55.20%	22.40%	22.40%	
**There is little evidence base for preconception care:**	Age 18-27:	21.10%	51.20%	27.70%	0.002

	Age 28-60:	13.40%	42.70%	43.90%	
**My GP is an appropriate person to offer preconception advice:**	Age 18-27:	74.10%	19.30%	6.60%	0.017
	Age 28-60:	67.30%	14.50%	18.20%	
**My practice nurse or community midwife is an appropriate person to offer preconception advice:**	Age 18-27:	76.40%	19.40%	4.20%	0.508
	Age 28-60:	76.40%	15.20%	8.50%	
**Friends and family are appropriate people to offer preconception advice:**	Age 18-27:	26.30%	41.90%	31.70%	0.056
	Age 28-60:	23.00%	32.10%	44.80%	
**The internet is an appropriate place to get preconception advice:**	Age 18-27:	35.30%	29.90%	34.70%	0.298
	Age 28-60:	41.60%	27.10%	31.30%	

Table S2. Results by parental status (i.e., preconception vs interconception) for questions on attitudes towards preconception health and care.

		**Agree**	**Neither Agree or Disagree**	**Disagree**	**Statistical test**
**Preconception care does not have any effect on pregnancy outcome:**	Has children:	12.80%	9.20%	78.00%	0.165
	No children:	7.10%	11.30%	81.60%	
**Preconception care is an important health issue for women of childbearing age:**	Has children:	88.60%	7.90%	3.60%	0.060
	No children:	79.20%	16.50%	4.20%	
**Preconception care is an important health issue for men of childbearing age:**	Has children:	64.50%	23.40%	12.10%	0.295
	No children:	58.00%	28.60%	13.40%	
**A dedicated clinic for preconception care is something I would make use of, if available:**	Has children:	45.40%	31.20%	23.40%	0.228
	No children:	54.20%	23.90%	21.80%	
**My friends/family do not plan for pregnancy:**	Has children:	10.70%	29.30%	60.00%	<0.001
	No children:	21.80%	37.00%	41.20%	
**Preconception care is a high priority for me:**	Has children:	53.60%	27.90%	18.60%	0.009

	No children:	43.30%	25.20%	31.50%	
**There is little evidence base for preconception care:**	Has children:	15.70%	37.10%	47.10%	0.011
	No children:	17.40%	53.00%	29.70%	
**My GP is an appropriate person to offer preconception advice:**	Has children:	70.00%	12.90%	17.10%	0.185
	No children:	71.00%	20.60%	8.40%	
**My practice nurse or community midwife is an appropriate person to offer preconception advice:**	Has children:	79.30%	11.40%	9.30%	0.951
	No children:	73.80%	21.90%	4.20%	
**Friends and family are appropriate people to offer preconception advice:**	Has children:	20.70%	35.70%	43.60%	0.074
	No children:	27.20%	37.70%	35.10%	
**The internet is an appropriate place to get preconception advice:**	Has children:	40.40%	27.70%	31.90%	0.693
	No children:	36.40%	32.20%	31.40%	

Supplementary Material S3.

Table S3. Results by age for questions on preconception health beliefs

		Strongly Agree	Agree	Neither Agree or Disagree	Disagree	Strongly Disagree	Statistical test:
**It is important for women to consume a healthy diet preconception.**	Age 18-27	36.50%	47.90%	8.40%	3.00%	4.20%	0.412
	Age 28-60	41.60%	43.40%	9.00%	3.60%	2.40%	
**It is important for men to consume a healthy diet preconception.**	Age 18-27	18.60%	42.50%	23.40%	12.60%	3.00%	0.001
	Age 28-60	32.10%	46.10%	13.90%	4.20%	3.60%	
**It is important for women to take folic acid preconception.**	Age 18-27	32.30%	21.00%	35.90%	7.80%	3.00%	<0.001
	Age 28-60	68.10%	18.70%	6.00%	3.60%	3.60%	
**It is important for women to take vitamin D preconception.**	Age 18-27	19.2%	39.50%	32.30%	6.60%	2.40%	0.662
	Age 28-60	25.90%	28.90%	38.00%	4.80%	2.40%	
**It is important for men to take vitamins preconception, such as vitamin D.**	Age 18-27	4.80%	25.10%	49.10%	16.20%	4.80%	0.114
	Age 28-60	11.50%	24.80%	47.90%	9.10%	6.70%	

**It is important for women to achieve a healthy weight preconception.**	Age 18-27	44.30%	39.50%	9.60%	3.60%	3.00%	0.452
	Age 28-60	38.00%	42.80%	13.30%	4.20%	1.80%	
**It is important for men to achieve a healthy weight preconception.**	Age 18-27	17.40%	39.50%	24.00%	14.40%	4.80%	0.103
	Age 28-60	21.80%	41.80%	21.80%	12.70%	1.80%	
**It is important for women to be physically active preconception.**	Age 18-27	23.40%	54.50%	11.40%	8.40%	2.40%	0.549
	Age 28-60	25.30%	51.20%	17.50%	4.20%	1.80%	
**It is important for men to be physically active preconception.**	Age 18-27	14.40%	45.50%	22.80%	14.40%	3.00%	0.11
	Age 28-60	18.20%	47.30%	23.60%	9.10%	1.80%	

Table S4. Results by parental status (i.e., preconception vs interconception) for questions on preconception health beliefs

		**Strongly Agree**	**Agree**	**Neither Agree or Disagree**	Disagree	Strongly Disagree	Statistical test:
**It is important for women to consume a healthy diet preconception.**	Has children	39.00%	43.30%	9.90%	3.50%	4.30%	0.539
	No children	39.70%	45.60%	7.90%	3.80%	2.90%	
**It is important for men to consume a healthy diet preconception.**	Has children	28.60%	44.30%	15.00%	7.90%	4.30%	0.253
	No children	23.40%	41.80%	20.90%	11.30%	2.50%	
**It is important for women to take folic acid preconception.**	Has children	68.10%	18.40%	6.40%	2.80%	4.30%	<0.001
	No children	40.60%	22.60%	28.00%	6.30%	2.50%	
**It is important for women to take vitamin D preconception.**	Has children:	19.90%	34.00%	38.30%	5.70%	2.10%	0.444
	No children:	23.00%	36.00%	32.60%	6.30%	2.10%	
**It is important for men to take vitamins preconception, such as vitamin D.**	Has children:	6.40%	22.70%	53.20%	11.30%	6.40%	0.608
	No children:	7.60%	25.20%	47.90%	14.70%	4.60%	
**It is important for women to achieve a healthy weight preconception.**	Has children:	32.60%	46.10%	13.50%	5.00%	2.80%	0.051

	No children:	44.40%	38.90%	11.30%	3.30%	2.10%	
**It is important for men to achieve a healthy weight preconception.**	Has children:	16.40%	42.10%	25.70%	12.90%	2.90%	0.797
	No children:	19.20%	38.10%	23.00%	16.30%	3.30%	
**It is important for women to be physically active preconception.**	Has children:	22.00%	48.90%	22.00%	5.00%	2.10%	0.771
	No children:	24.70%	51.90%	10.90%	10.50%	2.10%	
**It is important for men to be physically active preconception.**	Has children:	15.00%	43.60%	29.30%	10.00%	2.10%	0.731
	No children:	16.30%	44.80%	19.70%	16.70%	2.50%	

Supplementary Material S4.

Table S5. Ranking of preconception behaviours for women and for men by women without children (i.e., preconception).

**Preconception Practice for Women**	**Overall Ranking**	**Mean (SD)**	**Preconception Practice for Men**	**Overall Ranking**	**Mean (SD)**
Stop smoking cigarettes	1	3.48 (2.87)	Stop smoking cigarettes	1	3.0 (2.36)
Consume a healthy diet	2	4.17 (2.30)	Consume a healthy diet	2	4.10 (1.95)
Address mental health issues	3	5.84 (3.35)	Achieve a healthy weight	3	4.79 (2.33)
Achieve a healthy weight	4	5.89 (2.85)	Visit a healthcare professional	4	5.65 (3.74)
Take folic acid	5	6.46 (3.79)	Be physically active	5	5.92 (2.39)
Visit a health care professional	6	6.75 (4.01)	Address mental health issues	6	5.92 (3.29)
Cut out alcohol	7	6.85 (3.45)	Stop using e-cigarettes	7	6.29 (3.22)
Actively reduce/manage stress	8	6.92 (2.95)	Cut out alcohol	8	6.33 (3.24)
Stop using e-cigarettes	9	7.04 (3.35)	Actively reduce stress	9	6.90 (2.51)
Be physically active	10	7.08 (2.72)	Take vitamin D	10	8.06 (2.54)
Take vitamin D	11	8.46 (2.80)	Get adequate amounts of sleep	11	9.04 (2.02)
Get adequate amounts of sleep	12	9.05 (2.56)			

Table S6. Ranking of preconception behaviours for women and for men by women with children (i.e., interconception).

Preconception Practice for Women	Overall Ranking	Mean (SD)	Preconception Practice for Men	Overall Ranking	Mean (SD)
Stop smoking cigarettes	1	2.65 (2.30)	Stop smoking cigarettes	1	2.30 (1.45)
Take folic acid	2	3.59 (2.53)	Consume a healthy diet	2	2.82 (1.81)
Consume a healthy diet	3	4.51 (1.96)	Achieve a healthy weight	3	5.15 (2.20)
Stop using e-cigarettes	4	5.47 (3.25)	Stop using e-cigarettes	4	5.33 (2.61)
Achieve a healthy weight	5	5.64 (2.63)	Be physically active	5	5.52 (2.58)
Cut out alcohol	6	7.02 (3.26)	Cut out alcohol	6	6.33 (2.86)
Address mental health issues	7	7.09 (3.31)	Actively reduce/manage stress	7	7.06 (2.61)
Take vitamin D	8	7.25 (2.49)	Address mental health issues	8	7.39 (3.0)
Be physically active	9	7.44 (2.66)	Take vitamin D	9	7.42 (2.46)
Actively reduce/manage stress	10	8.09 (2.57)	Visit a healthcare professional	10	8.24 (3.47)
Visit a healthcare professional	11	9.32 (3.40)	Get adequate amounts of sleep	11	8.42 (2.26)
Get adequate amounts of sleep	12	9.94 (2.10)			

Table S7. Ranking of preconception behaviours for women and for men by women aged 18-27 years.

Preconception Practice for Women	Overall Ranking	Mean (SD)	Preconception Practice for Men	Overall Ranking	Mean (SD)
Stop smoking cigarettes	1	3.70 (2.94)	Stop smoking cigarettes	1	2.92 (2.44)
Consume a healthy diet	2	4.22 (2.38)	Consume a healthy diet	2	4.22 (1.85)
Address mental health issues	3	5.49 (3.32)	Achieve a healthy weight	3	5.22 (2.33)
Achieve a healthy weight	4	6.0 (2.94)	Visit a healthcare professional	4	5.31 (3.76)
Visit a healthcare professional	5	6.02 (3.86)	Address mental health issues	5	5.47 (3.41)
Actively reduce/manage stress	6	6.61 (3.01)	Cut out alcohol	6	6.11 (3.22)
Cut out alcohol	7	6.65 (3.42)	Be physically active	7	6.19 (2.25)
Stop using e-cigarettes	8	6.94 (3.39)	Stop using e-cigarettes	8	6.67 (3.27)
Take folic acid	9	7.23 (3.79)	Actively reduce/manage stress	9	6.78 (2.50)
Be physically active	10	7.29 (2.83)	Take vitamin D	10	8.28 (2.37)
Get adequate amounts of sleep	11	8.89 (2.50)	Get adequate amounts of sleep	11	8.83 (2.31)
Take vitamin D	12	8.96 (2.54)			

Table S8. Ranking of preconception behaviours for women and for men by women aged 28-60 years.

Preconception Practice for Women	Overall Ranking	Mean (SD)	Preconception Practice for Men	Overall Ranking	Mean (SD)
Stop smoking cigarettes	1	2.61 (2.14)	Stop smoking cigarettes	1	2.79 (1.76)
Take folic acid	2	3.68 (2.62)	Consume a healthy diet	2	2.85 (1.87)
Consume a healthy diet	3	4.41 (2.01)	Achieve a healthy weight	3	4.91 (2.24)
Achieve a healthy weight	4	5.63 (2.62)	Stop using e-cigarettes	4	5.3 (2.38)
Stop using e-cigarettes	5	5.92 (3.32)	Be physically active	5	5.45 (2.58)
Cut out alcohol	6	7.04 (3.34)	Cut out alcohol	6	6.18 (2.70)
Be physical active	7	7.09 (2.57)	Take vitamin D	7	6.97 (2.52)
Take vitamin D	8	7.11 (2.67)	Actively reduce/manage stress	8	6.97 (2.71)
Address mental health issues	9	7.20 (3.34)	Address mental health issues	9	7.76 (2.94)
Actively reduce/manage stress	10	8.06 (2.50)	Visit a healthcare professional	10	8.18 (3.87)
Visit a healthcare professional	11	9.50 (3.33)	Get adequate amounts of sleep	11	8.64 (2.19)
Get adequate amounts of sleep	12	9.75 (2.22)			
